# Intra-oral 3-D versus conventional miniplate in the treatment of mandibular symphysis and parasymphysis fracture

**DOI:** 10.6026/973206300211709

**Published:** 2025-05-31

**Authors:** Jyoti Biradar, Kishan Dudhat, Sanjay Byakodi, Amit Basannavar, Swapnil Shinde, Harshwardhan Kadam

**Affiliations:** 1 Department of Oral and Maxillofacial Surgery, Bharati Vidyapeeth (Deemed to be University) Dental College and Hospital, Sangli, Maharashtra, India

**Keywords:** Mandibular fractures, 3D titanium miniplates, conventional miniplates, occlusion, stability, radiographic healing, trauma, fixation techniques

## Abstract

Mandibular fractures, a common subset of maxillofacial trauma, have significant functional, esthetic, and psychological implications. Hence, 34 patients were divided into two groups: Group A (3D miniplates) and Group B (conventional miniplates). Results showed that both plating systems provided comparable outcomes in terms of occlusal correction, fracture stability, and radiographic healing by the third postoperative month, with no statistically significant differences. However, 3D miniplates offered advantages such as reduced surgical time and simultaneous stabilization of both superior and inferior fracture borders.

## Background:

Trauma is generally described as "a physical force that results in injury." Injuries to the maxillofacial area hold significant clinical importance due to their impact on both function and esthetics. On almost all occasion, there is a psychological aspect associated with such injuries, stemming from the patients' concerns about potential permanent scarring and subsequent facial deformities [1, 2]. The mandible has 13 muscle attachments, many of which accommodate various groups responsible for jaw closure (including the temporalis, masseter and medial pterygoid muscles), jaw opening (such as the digastric and lateral pterygoid muscles) and glottic function (including the genioglossus and geniohyoid muscles). Other muscles, like the buccinator, platysma, mentalis, mylohyoid, depressor labii inferioris and depressor anguli oris, also play roles in bone remodelling and displacement of fractures [3]. The inferior alveolar artery serves as the primary blood supply to the mandible, transitioning to the surrounding periosteum and muscle attachments as the body ages. The primary nerve supply to the mandible is the Inferior Alveolar Nerve, which innervates one half of the mandibular dentition exiting at the mental foramen to supply the soft tissues of the chin and lip. The lingual nerve runs inferior to alveolus and supra-periosteally in the third molar area [3]. A significant advancement in the treatment of mandibular fractures came with the development of the 3-D plating system by Farmand and Dupoirieux in 1992. The 3-D plates consist of two miniplates joined by interconnecting cross struts, effectively reducing torsional movement at the fracture site compared to single plates installed in tension areas [6].

The design of these plates allows for good blood supply to the bone due to the free area between the connecting arms. The stability provided by the geometric shape of 3-D miniplates surpasses that of standard miniplates, allowing for a reduction in thickness to 1mm, available in basic quadrangular forms with square or rectangular whole configurations, 3-D miniplates resist shearing, bending, and torsional forces, providing rigid stabilization at the fracture site. Their easy application avoids the need for time-consuming extraoral approaches and associated complications [6]. Additionally, their simplified adaptation to the bone minimizes fracture distortion or displacement, enabling simultaneous stabilization at both superior and inferior borders, saving time compared to conventional miniplates. However, one major drawback of 3-D plates is the difficulty in adaptation, requiring bending in three dimensions, which is more challenging than with 2-D plates [4- 5]. Therefore, it is of interest to evaluate intra-oral 3-D versus conventional miniplate in the treatment of mandibular symphysis and parasymphysis fracture.

## Materials and Methods:

The present prospective study was conducted to compare the effectiveness of 3D titanium miniplates with conventional 2mm titanium miniplates in the management of mandibular symphysis and parasymphysis fractures. The study was carried out in the Department of Oral and Maxillofacial Surgery at Bharati Vidyapeeth (Deemed to be University) Dental College and Hospital, Sangli, over a two-year period from April 2021 to April 2023. A total of 34 adult male and female patients diagnosed with mandibular symphysis or parasymphysis fractures and reporting to the department between March 2022 and March 2024 were included in the study. These patients were randomly divided into two equal groups. Group A consisted of 17 patients who were treated with 3D titanium miniplates, while Group B included 17 patients treated with conventional 2mm titanium miniplates. The clinical outcomes of Group A were compared with those of Group B to evaluate the differences in efficacy, stability and postoperative recovery between the two fixation methods.

## Inclusion criteria:

Adult, dentate patients requiring open reduction and internal fixation for mandibular Symphysis and Parasymphysis fractures.

## Exclusion criteria:

## Medically compromised patients:

[1] Edentulous and paediatric patients.

[2] Comminuted fracture of mandibular Symphysis and Parasymphysis.

[3] Patients refusing for follow-ups.

## Methodology:

All the patients in the study had undergone preoperative evaluation which included of complete case history, routine blood investigation, Chest X ray, Electrocardiogram and Orthopantomogram (OPG) / Three-Dimensional Computed Tomography (3D CT) face. Patients were also evaluated for other preoperative parameters *i.e.*, Stability (stable/unstable) Occlusion (Satisfactory or Deranged). Arch bars were placed preoperatively in cases with displaced parasymphysis or symphysis fracture. After preoperative work up including informed consent for General Anaesthesia and using 3-D / CTM patients were posted for surgery.

## Results:

In the present study, the control group (Group B), which was treated with conventional 2mm titanium miniplates, showed a mean outcome of 29.59 ± 7.76. In comparison, the study group (Group A), treated with 3D titanium miniplates, demonstrated a higher mean outcome of 34.76 ± 12.54. Statistical analysis using an independent t-test revealed a t-value of 1.448 and a p-value of 0.157. The control group (treated with conventional titanium miniplates) consisted of 16 males (94.1%) and 1 female (5.9%), while the study group (treated with 3D titanium miniplates) included 14 males (82.4%) and 3 females (17.6%). Occlusion was evaluated at multiple times intervals-pre-operatively, intra-operatively, at 1 week, 1 month and 3 months postoperatively-in both groups (Table 1). Pre-operatively, all patients in both groups (100%) presented with unstable fractures. As the condition was the same across all patients, no statistical comparison was applicable (Chi-square and p-value not available). Intra-operatively, a greater number of patients achieved stable fixation in both groups, with 64.7% stability in the control group and 70.6% stability in the study group. However, the difference between the groups was not statistically significant (Chi-square = 0.134, p = 0.714). At 1 week postoperatively, stability remained the same in both groups, with 64.7% of patients stable and 35.3% unstable. There was no difference in outcomes between the two groups and the result was again not statistically significant (Chi-square = 0.000, p = 1.000). By the 1-month follow-up, all patients in both groups had achieved 100% stability, which continued through the 3-month follow-up. Since the outcomes were identical in both groups, no statistical comparison was required at these intervals (Table 2). Radiographic assessment was performed at four different time intervals: pre-operatively, 1 week, 1 month and 3 months postoperatively to evaluate bone alignment and healing (Table 3). Pre-operatively, the majority of patients in both groups presented with displaced fractures: 88.2% in the control group and 76.5% in the study group. The difference was not statistically significant (Chi-square = 0.810, p = 0.368). At the 1-week follow-up, radiographic healing was found to be adequate in 76.5% of the control group and 82.4% of the study group. The difference in adequacy of alignment and healing was minimal and statistically insignificant (Chi-square = 0.180, p = 0.671). By 1 month, 94.1% of patients in both groups showed adequate radiographic healing. Only 1 patient in each group exhibited inadequate healing. There was no difference in outcomes (Chi-square = 0.000, p = 1.000). At the 3-month follow-up, 100% of patients in both groups showed adequate radiographic healing, indicating complete bone union. Since the results were identical, statistical comparison was not required.

## Discussion:

Any report concerning the study of mandibular fractures typically begins with a discussion on the historical background and the evolution of treatment methods. Documentation of mandible fractures dates back to 1650 BC, as evidenced by an Egyptian papyrus detailing the examination, diagnosis and treatment of such fractures and other surgical procedures. In the current study, we operated on 34 cases of maxillofacial trauma involving mandibular fractures that reported to either the Department of Oral and Maxillofacial Surgery, Bharati Vidyapeeth (Deemed University), Dental College and Hospital, Sangli or at Emergency Medicine Department, Bharati Vidyapeeth (Deemed University), Medical College and Hospital. They were treated 17 cases using open reduction and internal fixation with 3D miniplates and compared the outcomes with 17 cases treated using conventional miniplates. In our study, parasymphysis fractures accounted for the highest proportion (65%), followed by parasymphysis with angle fractures and then parasymphysis with condyle fractures (3%). Symphysis fractures constituted 10% of cases. In a study involving 191 patients with 280 mandibular fractures, the most common location was the angle region (28.21%), followed by Parasymphyseal fractures (21.07%) in terms of frequency [4, 6]. We found that the operating time for adapting and fixing the 3D plate was shorter compared to other methods. This aligns with findings from Raveh *et al.* [4, 5] and other researchers who also reported reduced operating time with 3D plates. The geometric configuration of the 3D plate, consisting of two horizontal bars interconnected with two vertical bars, allows for simultaneous stabilization of the fracture at both the superior and inferior borders, saving time during plate fixation. Postoperative radiographic evaluations showed at 1 week post-operatively excellent reduction in 76.5% and 82.4% in control and study group respectively and at 1 month 94.1% cases shows excellent reduction.

Lauer *et al.* [5] utilized 3D plates for transoral endoscopic-assisted condylar fractures. The triangular shape of the plate provides internal stability due to its three-dimensional nature. In our study post-operatively at 1 week 64.7% cases showed excellent stability after fracture reduction and fixation in both groups and at 1 month and 3month all cases showed excellent stability in both groups. Nakamura *et al.* [6] and other researchers observed postoperative complications in 110 patients with mandibular fractures, including malocclusion (3.6%), exposure of miniplate (3.6%), delayed union (1.8%) and infection (1.0%). These complications may arise due to factors such as inadequate reduction and stabilization, treatment delay, presence of teeth in the fracture line and failure to administer antibiotics or in cases of alcohol or drug abuse. In our study, we observed postoperative infection in one patient (5.9%) an in Group A and one patient (5.9%) in Group B. However, occlusal discrepancies were noted during the postoperative period at 1 week in 5 patients in group-A and 3 patients in group -B and postoperative period at 1 month in 3 patients in group-A and 1 patient in group -B and after 3 months there was no any occlusal discrepancies in any patient, indicating favourable occlusion outcomes with three- dimensional plating. Post-operative IMF done for cases that show occlusal discrepancies. Wittenberg *et al.* [7] utilized 3-D plates in treating mandibular fractures and noted that these plates were easier to place intraorally. Their closed quadrangular geometric shape and the ease of contouring and adapting to bony fragments provided excellent stabilization in three dimensions, resulting in low morbidity and infection rates. However, a potential limitation of 3-D plates could be the presence of excessive implant material, particularly due to the additional vertical bars needed to counter torque forces. Additionally, in cases where the fracture line passes through the mental foramina, there may be challenges associated with placement.

## Conclusion:

Overall, three-dimensional plating offers numerous advantages, including good intraoperative and postoperative stability without displacement or occlusal derangement. Additionally, it can lead to reduced costs and operating time, as no special armamentarium is r equired for its placement.

## Figures and Tables

**Table 1:** Comparison of occlusion at different time intervals between groups

**Time Interval**	**Occlusion Status**	**Control Group (n=17)**	**Study Group (n=17)**	**Total (n=34)**	**Chi-square Value**	**p-value**
Pre-operative	Deranged	16 (94.1%)	13 (76.5%)	29 (85.3%)	2.11	0.146
	Satisfactory	1 (5.9%)	4 (23.5%)	5 (14.7%)		
Intra-operative	Deranged	1 (5.9%)	0 (0.0%)	1 (2.9%)	1.03	0.31
	Satisfactory	16 (94.1%)	17 (100.0%)	33 (97.1%)		
1 Week	Mildly Deranged	5 (29.4%)	3 (17.6%)	8 (23.5%)	0.654	0.419
	Satisfactory	12 (70.6%)	14 (82.4%)	26 (76.5%)		
1 Month	Mildly Deranged	3 (17.6%)	1 (5.9%)	4 (11.8%)	1.133	0.287
	Satisfactory	14 (82.4%)	16 (94.1%)	30 (88.2%)		
3 Months	Satisfactory	17 (100.0%)	17 (100.0%)	34 (100.0%)	NA	NA

**Table 2:** Comparison of stability at different time intervals between groups (chi-square test)

**Time Interval**	**Stability Status**	**Control Group (n=17)**	**Study Group (n=17)**	**Total (n=34)**	**Chi-square Value**	**p-value**
Pre-operative	Unstable	17 (100.0%)	17 (100.0%)	34 (100.0%)	NA	NA
Intra-operative	Stable	11 (64.7%)	12 (70.6%)	23 (67.6%)	0.134	0.714
	Unstable	6 (35.3%)	5 (29.4%)	11 (32.4%)		
1 Week	Stable	11 (64.7%)	11 (64.7%)	22 (64.7%)	0	1
	Unstable	6 (35.3%)	6 (35.3%)	12 (35.3%)		
1 Month	Stable	17 (100.0%)	17 (100.0%)	34 (100.0%)	NA	NA
3 Months	Stable	17 (100.0%)	17 (100.0%)	34 (100.0%)	NA	NA
Note: p < 0.05 - Significant (*),
p < 0.001 - Highly Significant (**)

**Table 3:** Comparison of radiographic assessment at different time intervals between groups (chi-square test)

**Time Interval**	**Assessment Status**	**Control Group (n=17)**	**Study Group (n=17)**	**Total (n=34)**	**Chi-square Value**	**p-value**
Pre-operative	Displaced	15 (88.2%)	13 (76.5%)	28 (82.4%)	0.81	0.368
	Undisplaced	2 (11.8%)	4 (23.5%)	6 (17.6%)		
1 Week	Adequate	13 (76.5%)	14 (82.4%)	27 (79.4%)	0.18	0.671
	Inadequate	4 (23.5%)	3 (17.6%)	7 (20.6%)		
1 Month	Adequate	16 (94.1%)	16 (94.1%)	32 (94.1%)	0	1
	Inadequate	1 (5.9%)	1 (5.9%)	2 (5.9%)		
3 Months	Adequate	17 (100.0%)	17 (100.0%)	34 (100.0%)	NA	NA
Note: p < 0.05 - Significant (*),
p < 0.001 - Highly Significant (**)
